# Introduction of Octadecyl-Bonded Porous Particles in 3D-Printed Transparent Housings with Multiple Outlets

**DOI:** 10.1007/s10337-022-04156-w

**Published:** 2022-06-22

**Authors:** Liana S. Roca, Theodora Adamopoulou, Suhas H. Nawada, Peter J. Schoenmakers

**Affiliations:** Van ’t Hoff Institute for Molecular Sciences, Science Park 904, 1098 XH Amsterdam, The Netherlands

**Keywords:** Additive manufacturing, Microfluidics, Packing, Flow distribution, Flow control, Separation devices

## Abstract

Microfluidic devices for comprehensive three-dimensional spatial liquid chromatography will ultimately require a body of stationary phase with multiple in- and outlets. In the present work, 3D printing with a transparent polymer resin was used to create a simplified device that can be seen as a unit cell for an eventual three-dimensional separation system. Complete packing of the device with 5-μm C18 particles was achieved, with reasonable permeability. The packing process could be elegantly monitored from the pressure profile, which implies that optical transparency may not be required for future devices. The effluent flow was different for each of the four outlets of the device, but all flows were highly repeatable, suggesting that correction for flow-rate variations is possible. The investigation into flow patterns through the device was supported by computational-fluid-dynamics simulations. A proof-of-principle separation of four standard peptides is described, with mass-spectrometric detection for each of the four channels separately.

**Supplementary Information:**

The online version contains supplementary material available at 10.1007/s10337-022-04156-w.

## Introduction

Liquid chromatography is a versatile technique that is used to analyze samples from many fields, such as proteomics, metabolomics, lipidomics, etc. [1–3]. One of the common characteristics of life-science samples is the high degree of complexity, which necessitates a high peak capacity to fully resolve all components [4]. The introduction of ultra-high-performance liquid chromatography (UHPLC) allows the use of higher pressures, viz., longer columns and/or smaller particle diameters. In combination with shallow gradients, such systems have shown great promise in increasing resolving power [5]. However, a maximum peak capacity of 1400–1600 is predicted for one-dimensional (1D) LC [6], and a random distribution of peaks implies that only about 37% of the full peak capacity of a system can be realized [7]. To fully resolve life-science samples, higher peak capacities are needed. These can be achieved with multi-dimensional separations.

Multidimensional LC, employing orthogonal separation mechanisms in the different dimensions, promises much greater separation power. Ideally, the total peak capacity of the system will be the product of the peak capacities in each dimension [8]. In this way, a higher peak capacity can be obtained without a great increase in measurement time [9]. Multidimensional LC can be achieved in time-based separations by coupling different columns or in spatial separations [10]. The latter provides parallel, simultaneous separation in all but the first dimensions, which keeps the total analysis time much shorter and results in a much higher peak-production rate (peak capacity per unit time).

A design for a microfluidic device has been proposed previously [11]. The first-dimension (^1^D) separation takes place in a channel, the second-dimension (^2^D) separation in a perpendicular direction in a rectangular planar space (“flat bed”), and the third-dimension (^3^D) separation, again in a perpendicular direction, in a block. The analytes will be separated spatially in the ^1^D channel and ^2^D bed and eluted from the ^3^D block in the final separation. (“temporal” separation). Flow distributors are needed to transfer the analytes to the next dimensions and to create homogeneous flow in the ^2^D and ^3^D directions. To create such a device, 3D-printing techniques have been proposed and flow-control [12], implementation of stationary phases [13, 14], and detection methods all need to be considered for its operation. Given the current state of the art in 3D printing, we envisage devices slightly larger than that described before [11] to accommodate channels of between 1 and 2 mm internal diameter. Smaller devices are feasible with high-resolution 3D printing, such as two-photon polymerization [15] or hybrid stereolithography [16].

The stationary phase can be a packed bed (polymer or silica-based particles) or a monolith, with various surface functionalities. Monoliths are formed from a liquid mixture of monomers, cross-linkers, porogens, and initiator, which has the advantage that it can easily be introduced into any structure. The formation of the organic monolith can be performed by UV irradiation or thermal initiation. However, the polymerization process is highly exothermic [17]. This causes temperature differences in large spaces, with the centre getting warmer than the edges, leading to a heterogeneous bed. Particle-packed columns yield higher separation efficiencies and better reproducibility than organic monolithic column and the former are used much-more widely.

However, the introduction of particles in a device can present its own set of challenges. Procedures for the packing and consolidation of cylindrical columns are well established, and sources of packing heterogeneities have been the subject of several studies [18–21]. There are few published examples of packing channels in microfluidic devices [22, 23]. The demands of a spatial 2D or 3D device introduce their own set of considerations, compared to packing a cylindrical column with a single inlet and outlet, or a single channel in a microfluidic device. A cuboidal or block-shaped region, necessary for all dimensions, creates dead zones, where packing densities may be lower. The proposed devices also require flow distributors and/or collectors to distribute fluid flow homogeneously within the ^2^D and ^3^D spaces. The design of these flow distributors will also influence the homogeneity of the packed regions. An additional consideration is the 3D-printing method used to create the device, which can introduce surface features [24] not present in mechanically polished cylindrical columns. Nonetheless, it is highly relevant to attempt creating particle-based stationary phases, because of their suitability to larger aspect ratios needed for ^2^D and ^3^D regions.

In this project, we aimed to study the introduction of particles in a block-shaped region, akin to a possible third-dimension block in a 3D-spatial LC device. We sought a transparent material for 3D printing suitable for Reversed-Phase Liquid Chromatography (RPLC) separations that would allow the visualization of the packing and withstand the pressure needed for introducing the particles. Moreover, we aimed to develop a packing procedure, to test the properties of the packed device, and to produce a proof-of-principle separation.

## Materials and Methods

### Chemicals

Acetonitrile (ACN, MS grade) and 2-propanol (IPA, HPLC grade) were purchased from Biosolve Chimie (Dieuze, France). Milli-Q water (18.2 mΩ) was obtained from a purification system (Millipore, Bedford, MA, USA). C18 particles (5 µm diameter, 100 Å pre size) were purchased from Fuji Silica Chemical (Lausanne, Switzerland). An HPLC peptide-standard mixture was purchased from Sigma-Aldrich (Darmstadt, Germany). The methacrylate-based resin Formlabs Durable was purchased from Formlabs (Somerville, MA, USA).

A Next-Advance frit kit (Troy, NY, USA), fused-silica capillaries (200 µm ID, 360 µm OD) (CM scientific, Silsden, UK), PEEK tubing (IDEX, Lake Forest, IL, USA), and ferules, nuts, and unions (Vici-Valco, Houston, TX, USA) were used to prepare the connections.

### Instrumentation

For constructing the devices, the Form-2 3D-printer (Formlabs) and Form Cure chamber (405 nm wavelength; Formlabs) were used. Packing of the devices was performed using a Shimadzu LC-10AD VP pump (Shimadzu‘s Hertogenbosch, The Netherlands). The flow measurements were performed with an Agilent 1100 Series Pump (Agilent, Waldbronn, Germany). The LC–MS measurements were performed with Waters ACQUITY UPLC and Waters Synapt G2 (Waters Corporation, Milford, US).

### Design of Device and Printing

**Fig. 1:**
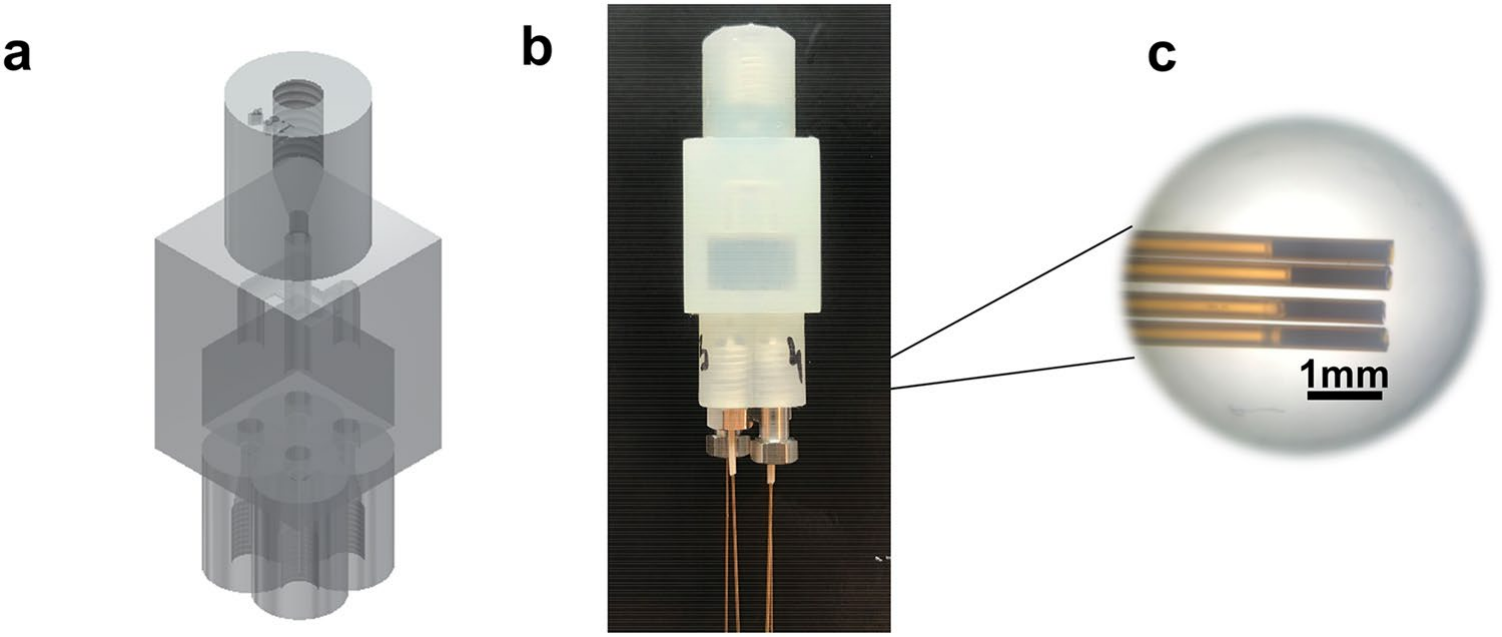
Design of the 3D-printed device (**a** CAD picture; **b** printed device). Fused-silica capillaries (200 µm ID) containing frits (Kasil) were used to contain the particles (**c**)

To study the efficacy of particle packing and the performance of the bed for a potential third dimension, devices with a flow distributor (FD) starting from one inlet, a 7 × 10 × 10 mm ^3^D space, and four outlets were studied. The initial device, shown in Fig. 1, comprised an FD with 1.4-mm ID channels and a wall thickness of 1 mm. In following iterations, the wall thickness was increased to 2.5 mm to enhance the pressure resistance of the devices. Additionally, the ID of the flow-distributor channels was adjusted from 1.4 to 2 mm to enhance the ease of packing. All devices were designed via Autodesk Inventor (Autodesk, San Rafael, CA, USA).

To visualize the anticipated influence of packing heterogeneities, computational-fluid-dynamics (CFD) simulations were performed. ANSYS Workbench Fluids and Structures Academic package (version 17.1) was used (ANSYS, Canonsburg, PA, USA). The examined case was discretized with ANSYS Meshing. The geometry was meshed with tetrahedral cells and inflation layers were used on the flow distributor. The total number of cells in this setup was 12,956,270. All simulations were conducted using the Fluent solver (ANSYS), solving for flow and species transport [25]. To simulate the effect of packing heterogeneities on the flow profile, a mixture of dye and water (1% dye) was injected from the ^3^D inlet and flushed with one device volume of water toward the ^3^D space. Two cases were examined, viz*.,* one with perfect packing of the whole device and one where a part of the FD was not fully packed. In both cases, the permeability was set at 10^–15^ m^2^. The value of the velocity at the device inlet was adjusted so as to obtain a velocity of 1 mm/s in the block-shaped region.

The device was fabricated through 3D printing, more specifically stereolithography. First, the design was converted to STL format and then loaded to PreForm (FormLabs software). Printing orientation and settings were optimized for high resolution and fabrication time, after which the form file was loaded to the Form-2 printer. After printing, post-processing of the parts was necessary (Fig. S5, Supplementary Information). This included sonication and flushing of channels with 2-propanol and compressed air to remove any uncured resin. Thereafter, the parts were placed in a Form Cure chamber (405 nm; Formlabs) for UV and thermal curing for 60 min at 60 °C.

For fluidic connections, straight threads (#10-32 UNC, major diameter 4.83 mm, thread pitch 0.794 mm or #6-32 UNC, major diameter 3.5 mm, and thread pitch 0.794 mm) were created using a hand tap. Conical ferrule seats were included in the designs of the devices to prevent leakage.

### Connections (frit)

Confinement of the particles inside the device needs a barrier permeable for the solvent, but not for the stationary-phase particles. In this project, the confinement was achieved by the use of silica frits. These were created inside fused-silica capillaries (200-μm internal diameter). A solution of Kasil 1624 (60 μL) and formamide (20 μL) was prepared and vortexed gently. The capillaries were dipped (1–2 s) in this mixture and then placed in an oven at 100 °C overnight. After the formation of the frit with 10 to 20 mm length, the capillary was cut so as to retain only 1 or 2 mm of monolith (Fig. 1C). On the frit side of the capillary, a sleeve and ferrule were added and the connection was made. Two sizes of nuts were used to allow for more space to tighten them (see Fig. 1B).

### Packing Procedure

A slurry of 0.8 g of particles in 4 mL of IPA/water 50/50 by volume (0.2 g/mL) was prepared and sonicated for 15 min. The slurry chamber was an empty cylinder (250 × 4.1 mm ID, 3.3 mL internal volume) with a piece of metal tubing (specified aa 0.04″ ID, 100 mm length) connected at the end to make the connection with the device. The setup was aligned vertically with the slurry chamber on top, followed by the metal tubing connected to the inlet of the 3D-printed device. The components were held in place using metal clamps. The slurry was introduced into the cylinder and the top was connected to a pump (Shimadzu LC-10AD VP pump). Packing was performed under constant flow (100 µL/min) and the pump was stopped when a sharp increase in pressure was observed and the pump pressure reached 40 bar (4 MPa). Higher pressures were not possible, due to the limited pressure resistance of the device (see Supplementary Information, Fig. S8).

### Characterization of the Packed Devices

The permeability of the devices was determined by measuring the pressure drop across the empty device and across the device after packing. When being flushed with water at various flow rates. Equation 1 was used to calculate the permeability [26]

1 $$ k = \frac{\mu \times L \times Q}{{A \times \Delta P}}, $$

where *k* is the permeability (m^2^), *µ* is the viscosity of the solvent (Pa × s × m), *L* is the length of the column (m), *Q* is the flow rate (m^3^/s), *A* is the surface area (m^2^), and ∆*P* is the difference in pressure drops between the empty device and the packed device (Pa).

The uniformity of the effluent flow was determined by flushing the packed device with water at 0.2 mL/min for 15 min. The effluent from each outlet was collected and weighted in triplicate. The total weight collected was expected to be 3 g and the weight collected from each outlet 0.75 g (25%). The average percentage collected from each outlet and the standard deviations were determined.

### Separation and MS Detection

Since the device contained four outlets, the backpressure caused by a connecting one of the outlet tubings to a detector would influence the flow through the device. Therefore, an MS detector was used that did not add any backpressure. The effluent flow from all each of the outlets was measured. The connection was made using a PEEK nut added to the outlet capillary, which was then connected to the inlet of the MS (Fig. S12, Supplementary Information). After that, the device was connected to the MS (Waters Synapt G2) and detection was performed individually after each of the four outlets, with the effluent flow measured for each of the three remaining outlets to verify that the MS did not affect the flow rates through the different channels.

The device was used under reversed-phase conditions to perform the separation of four peptides (Gly-Tyr, Val-Tyr-Val, Met-Enkephalin, and Leu-Enkephalin). A gradient from 15% ACN to 50% ACN (with a constant 0.1% of formic acid, FA, as additive) in 10 min was performed at 0.3 mL/min. The peptides were dissolved in water containing 2% ACN and 0.1% FA by volume (0.02 mg/mL of each peptide) and 5 µL were loaded on the device (0.1 µg of each peptide).

The MS method was set to negative mode, capillary voltage 1.8 kV, sampling cone 20 V, extraction cone 2 V, source temperature 110 °C, desolvation temperature 350 °C, cone gas 10 L/h, and desolvation gas 800 L/h. The mass range was 100–1200 Da, and only MS^1^ was acquired.

## Results and Discussion

### Design of the 3D-Printed Device

The 3D-spatial separation devices that we are aiming for consist of a first-dimension channel, a second-dimension planar separation space in a perpendicular direction, and a third-dimension separation block underneath the second dimension (Fig. S1 in supplementary information). The design of the present device is a simplification of the third-dimension space, intended to study possible packing techniques. Multiple aspects need to be considered when building a 3D separation block, so as to ensure flow confinement and added selectivity. The aspects of flow confinement have been investigated using simulations [12] and it was concluded that a permeability difference of two orders of magnitude is needed for a good flow confinement between the second and third dimensions. One possibility to achieve this may be a highly permeable monolith in the second-dimension separation space and a particle-packed bed for the third-dimension separation. Our simplified device, shown in Fig. 1, contains a flow distributor on top, a separation block, and four outlets at the bottom, which may be seen as an elementary unit of the ultimate separation device.

### Packing Considerations for 3D-Printed Devices

#### Solvent Used for Packing

When packing devices, a very important consideration is the suspension of the particles in the packing solvent. For C18 particles, due to their hydrophobic nature, organic solvents, such as methanol, iso-propanol (IPA), chloroform, acetone, etc*.*, may provide good, stable suspensions. In contrast, particles agglomerate and float on top when submerged in water (Fig. S5, Supplementary Information). The stability of suspensions is also enhanced by a higher solvent viscosity and a small density difference between solvent and particles. However, we also had to consider the solvent compatibility of the device. The material used for printing the devices (Formlabs durable) was not stable in methanol, acetone, or chloroform, and showed swelling when exposed for a long duration to pure IPA or acetonitrile (ACN) (see Fig. S4 in Supplementary Information).

IPA is the recommended solvent for post-processing of the 3D-printed devices (see “Design of Device and Printing”). Also, it has a relatively high viscosity and is miscible with water. Therefore, a mixture of IPA and water was thought to be a good solvent for packing. The C18 particles were suspended 50% IPA in water (by volume), which allowed for solvation of the particles and yielded a homogeneous slurry. Sonication of the slurry for 15 min was used to prevent any possible agglomeration of the particles. Thereafter, the slurry was added to the packing cylinder and a constant flow of 50% IPA was used for packing.

#### Pressure Resistance

Initially, the device was designed to have a wall thickness of 1 mm around the separation space. The device was connected to the LC pump with 150-µm ID connection tubing. The pressure of the system was measured at different flow rates (0.1–1.5 mL/min), without the device installed. After installing the device, we ramped up the flow-rate (starting at 0.1 mL/min) and thus the pressure. The devices typically functioned well until about 35 bar (3.5 MPa). At that point, the wall around the separation space would break and start to leak (see Fig. S3 in Supplementary Information). This was performed to see at what pressure the empty device would break or start leaking and which were the weak points.

**Fig. 2:**
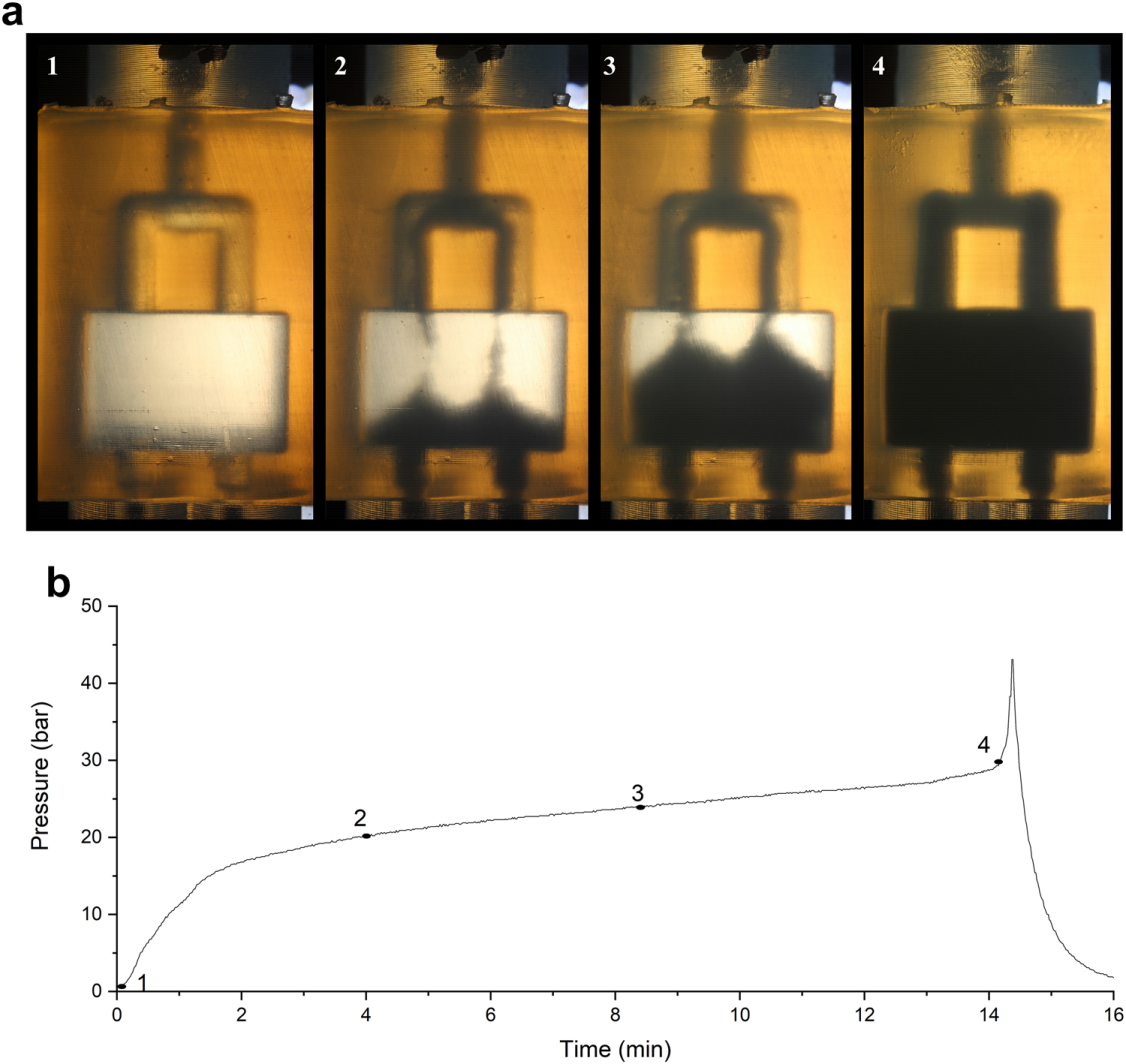
Illustration of the process of packing a device. **a** Visual observation at various stages of the process; **b** pressure profile during packing with the numbers corresponding to the pictures of (**a**). C18 particles 5 µm introduced as a slurry in 50% IPA

**Fig. 3:**
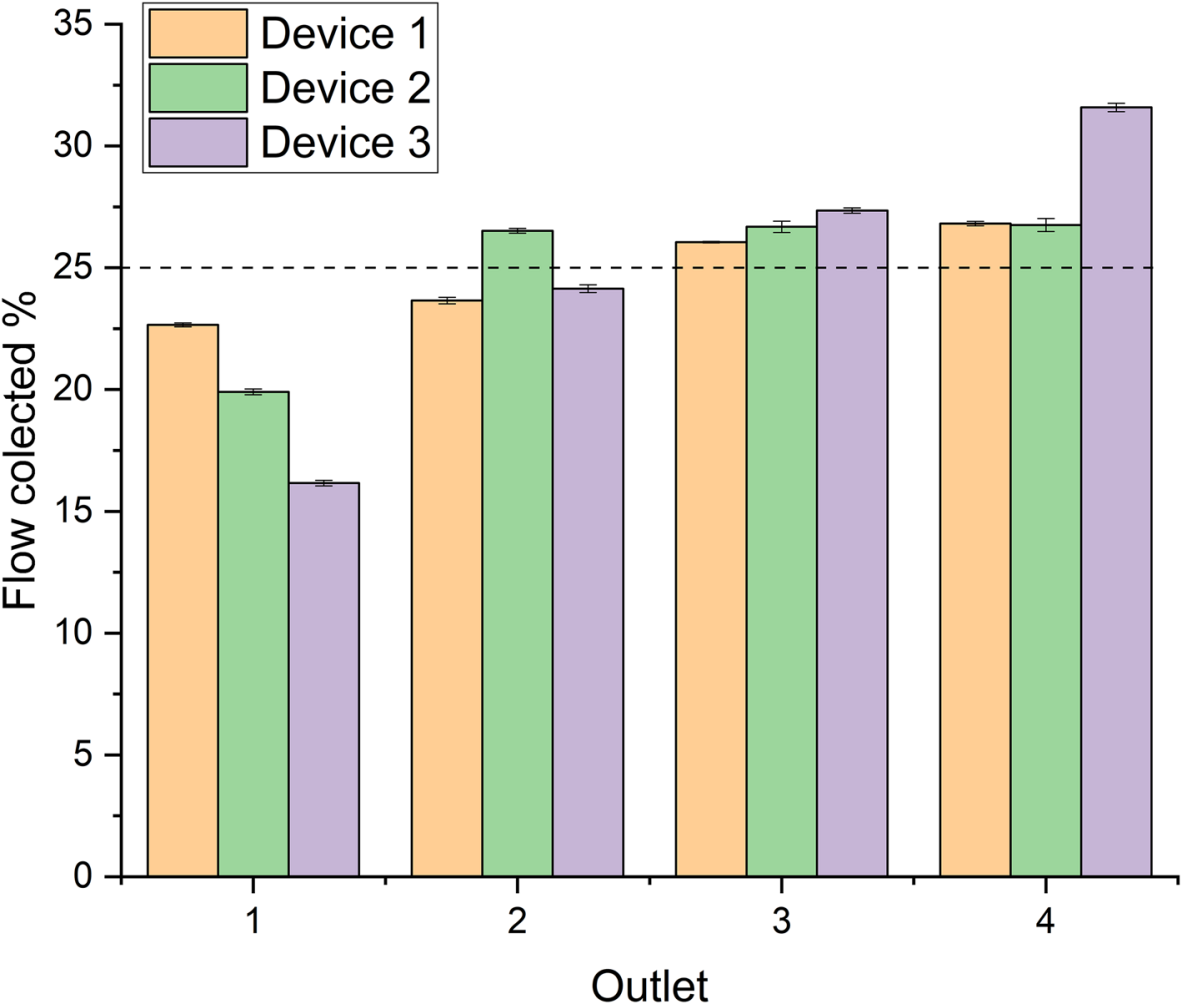
Relative effluent flow rates from all outlets of three different devices, measured by collecting and weighing. Measurements were performed in triplicate and the average value was plotted with the standard deviation. 25% from each outlet would represent an ideal situation

**Fig. 4:**
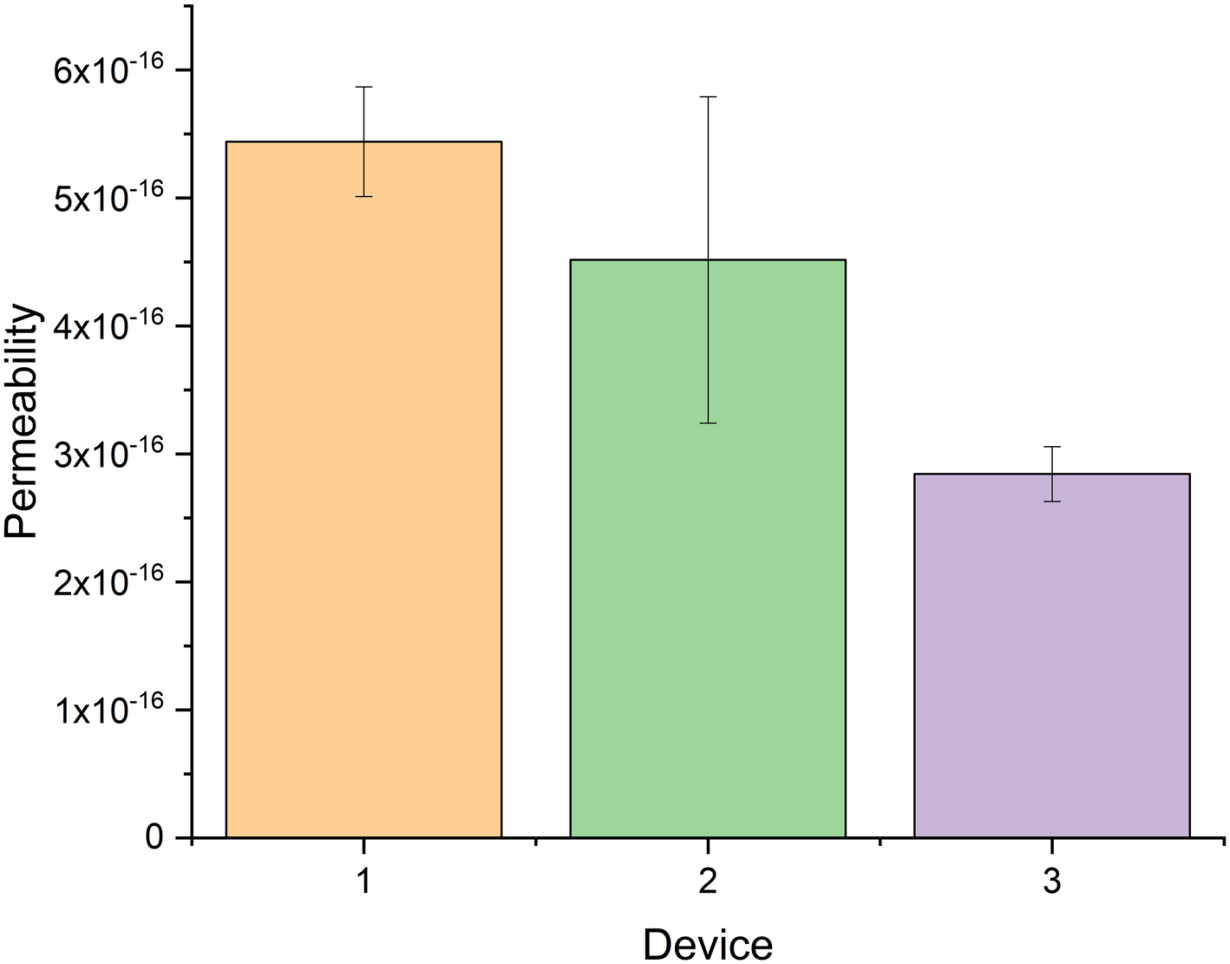
Permeability of three packed devices measured using water at 0.1, 0.2, and 0.4 mL/min. The average permeability and the standard deviation are plotted

The wall thickness was increased to 2.5 mm to improve the pressure resistance of the device. This still allowed for a good visualization of the separation space. The highest pressure achieved with this device was 80 bar for a short period of time (packed device). The vulnerable points in the device were the walls surrounding the empty block and the fittings. A further increase in wall thickness was deemed undesirable, as this would reduce the transparency of the device. At a flow rate of 1.1 mL/min, the pressure drop was dominated by the system and connections. The empty device proved stable at this flow rate and showed a fairly constant pressure of 30–40 bar. Only after prolonged operation at higher pressure cracks around the separation space or leaks from the connections were observed. Therefore, pressures should not exceed 40 bar if at all possible. While the increased wall thickness increased the pressure resistance of the device, swelling of the packing chamber was not completely eliminated, as seen in Fig. 2—4.

Another cause of failure of the printed devices after repeated use was the erosion of the printed substrate in the threaded ports. PEEK fittings (UNF 10-32) at the inlet of the device were found to be less damaging when attached and removed repeatedly, compared to metal fittings. PEEK fittings (UNF 6-32) were also tried at the outlets, but the nut did not tighten enough to hold the capillaries in place. Metal fittings were used at the outlets instead. When the fittings started leaking the device could not be used anymore.

#### Packing Procedure

The packing procedure was monitored visually (Fig. 2A) and by monitoring the backpressure (Fig. 2B). Initially (region 1), the outlet zones were packed, resulting in a steep rise in the pressure. This was followed by a gradual increase, while the main region was being packed (zones 2 and 3). The flow-rate was chosen so as to have a reasonable backpressure while packing. Most devices had a pressure around 20 bar for the main part of the packing procedure at a constant-flow rate (0.2 mL/min; see Fig. 2B). When the flow distributor and the metal tubing connecting the device to the packing cylinder also started to be filled with particles, the pressure increased rapidly and the device was considered to be fully packed (region 4 in Fig. 2B). At this point, the flow was stopped.

For six devices that were packed without any interruption, the average packing pressure was 21.5 bar and the average time it took until the devices were fully packed was 13.7 min. Using this procedure to monitor the packing, transparency of the device is not a prerequisite. Packing of non-transparent devices (e.g., metal printed devices) may also be performed by monitoring the pressure during packing. This would remove the limitation on compatible solvents currently encountered and increase the maximum pressure during packing and operation. However, the design of the connections should be adapted and the effects of the surface roughness should be investigated.

Sonication during packing was also attempted. It has been shown for capillary columns that using sonication can prevent particle aggregation, hence column clogging, and can make the packing process faster, ensuring the stability of the slurry suspension [27]. We observed that the packing pressure was lower during sonication, probably because the bed was not consolidating until the end of the process. However, the observed permeability was comparable to that observed without sonication, while the uniformity of flow output was slightly worse (see Figs. S9 and S10 in Supplementary Information). Moreover, due to the sonication, the outlet nuts moved, loosening the connections and allowing particles to escape around the ferrule and into the threads. Therefore, sonication was not considered a valuable addition to the packing of the devices.

### Characterization of Packed Devices

The devices packed with C18 particles were characterized by measuring the effluent flow from each outlet, the permeability, and by the separation of peptides under gradient conditions.

#### Flow Uniformity

During experimental testing, measurements were performed in triplicate to determine the variability in effluent flow from each outlet and the variability between the outlets. In Fig. 3 the average relative flow collected is illustrated for three devices. The lowest flow recorded from any outlet was 15% (instead of the expected 25%), which indicates a large variability. However, the variability of individual outlets for the triplicate measurements was very low. This would suggest that a packed device with multiple channels may eventually be used in practice, provided that a flow marker is used to correct for the residence time in each channel. The added value for the separation power of the device can still be realized, even with deviations at the flow distribution, as long as the measurements are repeatable.

#### Permeability

The permeability of the devices was also considered as a measure of packing uniformity. The permeability was calculated by measuring the pressure drop across the packed device corrected for that across the empty device at a given flow rate. The permeability of three devices can be seen in Fig. 4. The average permeability was calculated using water at three different flow rates (0.1, 0.2, and 0.4 mL/min). Higher flow rates were not used to avoid high backpressures. The contribution of the flow distributor was not considered in the calculation of the permeability. Only the area and length of the separation space were used in Eq. 1 (see “Characterization of the Packed Devices”). Permeability values in the order of 10^–16^ were obtained. For a column packed with 5 µm particles, a permeability of 10^–14^ would be expected [28, 29]. From our results, it seems that the packed bed obtained was denser or that the flow distributor had a large impact on the permeability calculation. Considering the presence of particles in the channels of the flow distributor, the latter is considered more likely. This assumption can also be supported by a theoretical calculation of the influence of the packed flow distributor on the device pressure. We found that 94% of the backpressure is due to the flow distributor and only 2% due to the separation space (Supplementary Information Table S1).

In addition to radial heterogeneity (“Flow Uniformity”), the effect of axial heterogeneity on the local permeability was studied by applying a step gradient from 100% IPA to 100% water and recording the backpressure of the system. A device with a perfectly constant axial permeability is expected to lead to a linear decrease in the system backpressure from P_1_ (100% IPA) to P_2_ (100% Water), with slight non-linearity in the pressure profile being introduced by the packed flow distributor, device band broadening, and axial mixing.

**Fig. 5:**
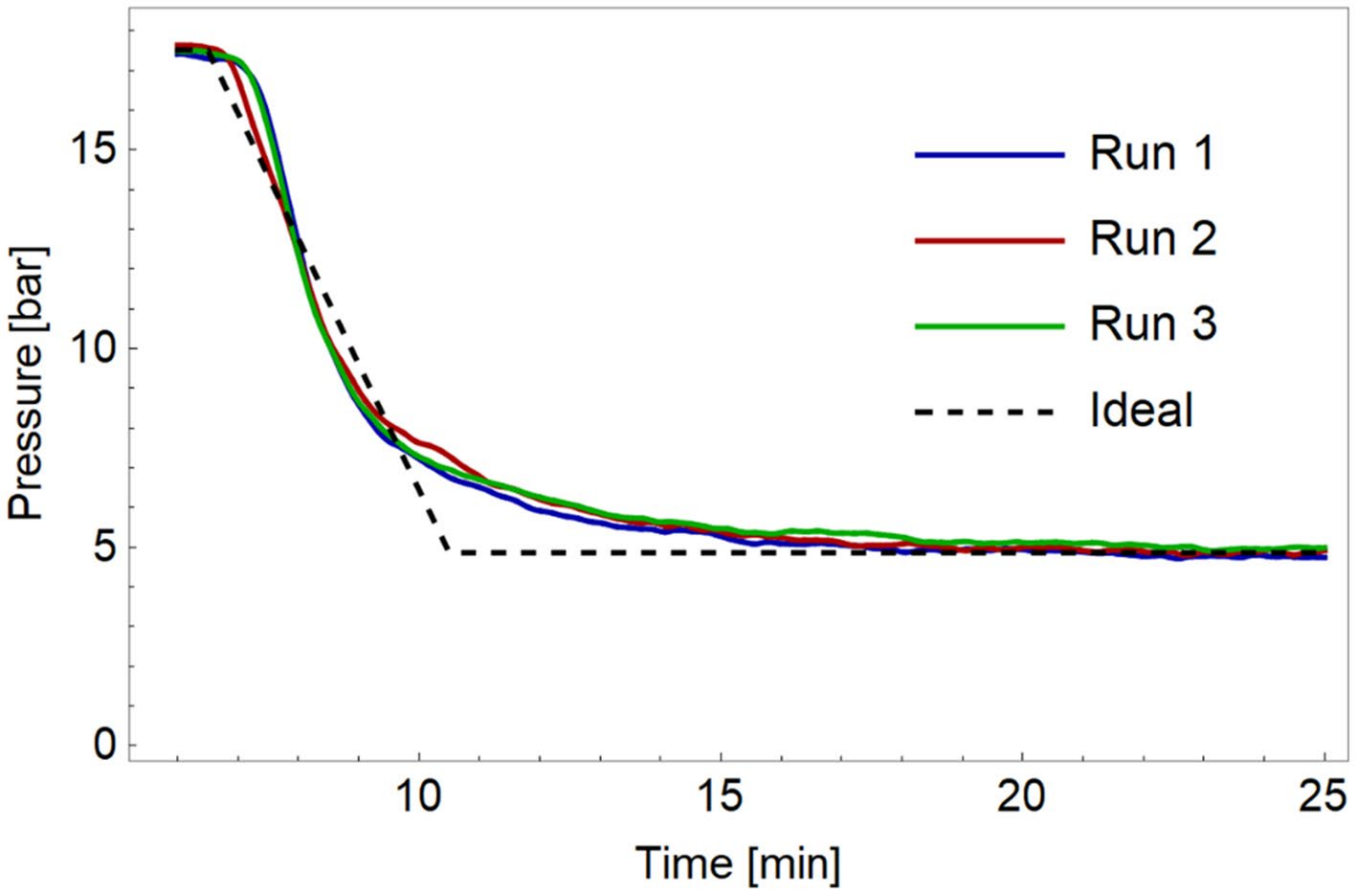
Pressure profile arising from a gradient from 100 IPA to 100% water in 0.1 min

A significantly curved pressure profile at the start of the gradient indicates under-packed or heterogeneously packed regions within the flow distributor and prominent tailing at the end of the pressure profile indicates heterogeneities in the separation region. The measured profiles shown in Fig. 5 show an initial steep decline (4–5 min) when the gradient passes the flow distributor. The curvature is relatively minor, which may be due to a homogeneous packing and/or the relatively small volume of the flow distributor. The steep decline is followed by a shallow part when the gradient passes through the separation region. The curvature of this part of the curve is indicative of heterogeneities in the packed bed.

#### Separation of Peptide Standards

The device was envisioned to be the third dimension of a separation space. We attempted the separation of four peptides under reversed-phase gradient conditions. The separation bed was only 7 mm in length and a gradient of 10 min was programmed. The effluent of each outlet had to be measured individually, since a multi-channel detector was not available. To obtain correct results, a detector was needed that did not alter the flow distribution between the four outlets.

Initially, the flow output of the device was recorded in each outlet, and then, one of the outlets was connected to a mass spectrometer (MS). The flow output from the three remaining channels was measured again to determine the influence of the MS on the flow profile. A comparison between the free outlets and one outlet connected to the MS can be seen in the supplementary information (Fig. S11 and Table S2). We observed no change in backpressure caused by the MS and no influence on the flow distribution across the four channels. Therefore, the MS was deemed a good option for detection.

**Fig. 6:**
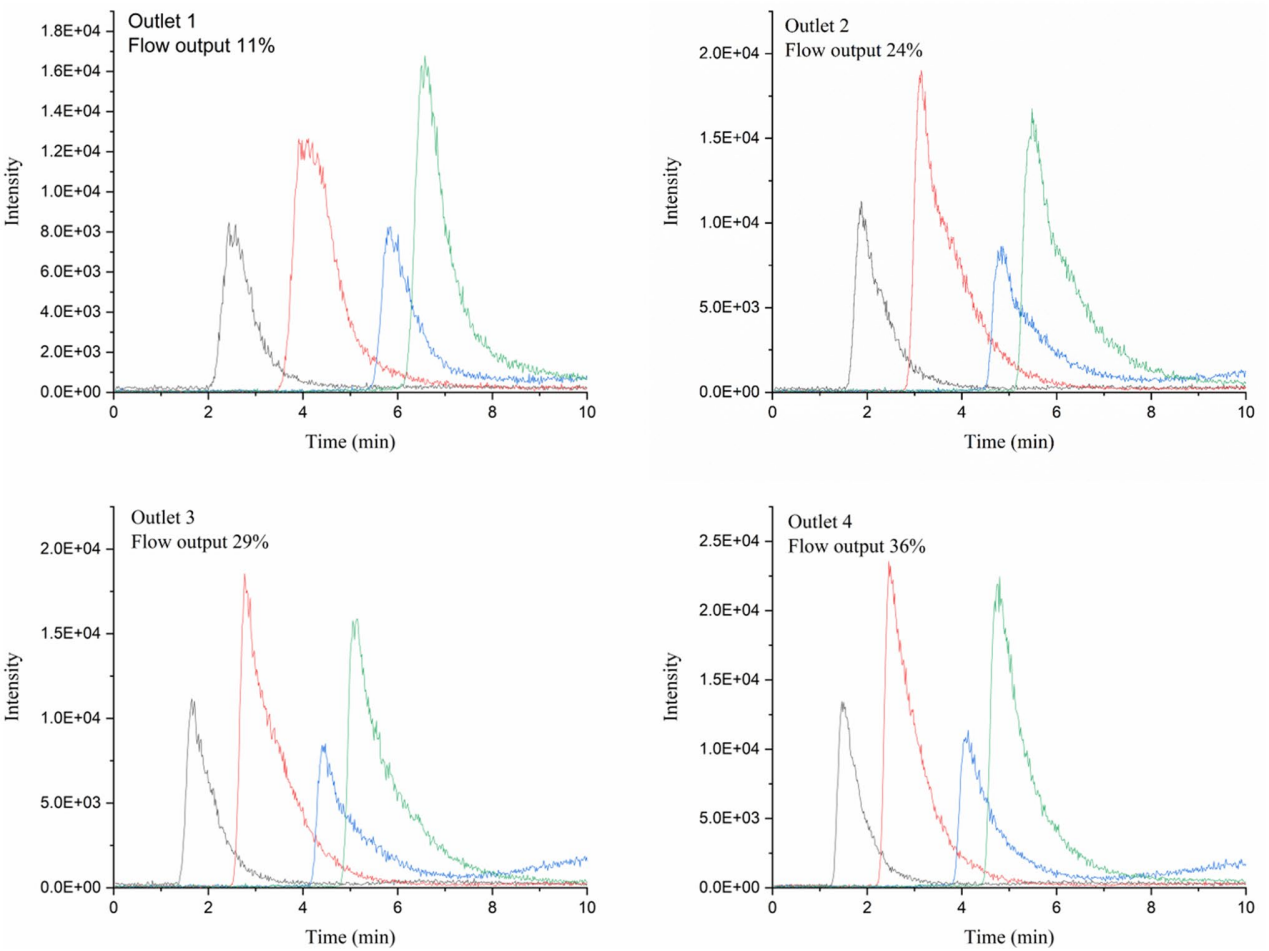
Separation of four peptides using a gradient from 15 to 50% ACN in 10 min at a flow rate of 0.3 mL/min with MS detection at each of the 4 outlets. Peptides in order of elution: Gly-Tyr (black), Val-Tyr-Val (red), Met-Enkephalin (blue), and Leu-Enkephalin (green)

The separation of the peptide mixture can be seen in Fig. 6. The peptides separated were Gly-Tyr, Val-Tyr-Val, Met-Enkephalin, and Leu- Enkephalin (structures shown in Fig. S13, Supplementary Information). The elution order of the four peptides was the same in all four outlets, but a shift in retention time was observed, which could be correlated with the flow through each outlet. Outlet 4 exhibits the highest linear velocity. Therefore, the composition gradient will arrive earlier and be steeper, leading to faster elution and sharper peaks. Outlet 1 showed broader peaks and the longest retention times, due to a relative flow of only 11%. The peaks were not baseline separated. However, with MS detection, we were able to use extracted ion currents (EIC) to easily identify the four peptides. The repeatability of the separation was investigated for the fourth outlet. The same gradient was run three times and the EICs plotted as overlays (see Fig. S14 in Supplementary Information).

In all outlets, severe peak tailing was observed. This may possibly be attributed to dead zones in the corners of the cube. Even in a cylindrical column, it has been shown that the mobile-phase velocity can be 2–5% lower close to the wall [30]. When the separation space is a cube, the linear velocity in the corners may be even lower. This will then lead to distortion of the peaks. Such “wall effects” will have less influence on a device that features a larger array of outlets.

The peak capacity for this imperfect device was calculated based on Eq. 2 [31]

2 $$ n_{c} = \frac{{t_{g} }}{{1.7 \times w_{{\frac{1}{2}h}} }} + 1 $$

where *n*_*c*_ is the peak capacity, $w_{{\frac{1}{2}h}}$ is the peak width measured at half height (min), and *t*_*g*_ the gradient time (min). And a value of *n*_*c*_ = 9 was obtained.

In a perfect multi-dimensional system, the total peak capacity may be obtained by multiplying the peak capacities obtained in each dimension [11]. When using a limited number of channels, this puts a limit on the actual achievable peak capacity. Also, the effective peak capacity is limited by the orthogonality of the different separations. The time needed for the separation is determined by the sum of the analysis times in each dimension. Assuming a final device with 16 outlets between the first and second dimension (hence, an assumed ^1^D peak capacity of ^1^*n*_*c*_ = 16), and 16 outlets between each second-dimension channel and the third dimension (^2^*n*_*c*_ = 16), and with ^3^*n*_*c*_ = 9. The anticipated total peak capacity of the device would be 2304.

## Conclusions

A device was designed so as to mimic a unit in a three-dimensional separation space. Such devices were successfully created in this work using a Form-2 3D-printer (Formlabs) using Durable as resin, resulting in a good solvent stability, transparency, and flexibility. The latter property allowed connections to be made to fritted capillaries, providing a barrier for the particles. We succeeded in packing C18 porous particles suspended in 50% IPA under constant-flow conditions (0.2 mL/min). Monitoring of the pump pressure during packing was found to offer an indication of the completion of the packing process. Therefore, the procedure is also applicable to non-transparent devices, allowing the possibility to use of metal printing. The use of metal printing would allow for a wider range of organic solvents and the devices may withstand higher pressures.

When investigating the flow stability and uniformity through the device, highly repeatable measurements were obtained for each single outlet, but there were large variations between the four outlets. The latter could be caused by errors in printing (*e.g.,* unequal channels in the flow distributor), differences in the inner diameters of outlet capillaries, differences in length and/or permeability between different frits, or differences in particle consolidation. In spite of all this, the repeatability of the device promises future gains in separation power by adding a third-dimension separation.

A proof-of-principle separation of peptides using the device was obtained by loading the sample from the flow distributor. In future experiments, separations of samples will be developed in the plane above the separation space and different analytes will be sent to each outlet. In the present case, we obtained the same chromatogram from all the outlets with some variation in retention times, that could be correlated with differences in the flow output. The separation of the four peptides was repeatable, but peak tailing was observed due to dead zones in the device, most likely in the corners of the separation space. This situation may be improved by smoothing the corners of the separation space and by creating more outlets. The detection was performed for each outlet separately by connection to the MS. In the future, other detection methods will be employed to monitor all channels simultaneously or to store fractions of each outlet before detection (droplet collection). If droplet collection is employed, detection could then be performed by scanning (e.g., fluorescence spectroscopy) or matrix-assisted laser-desorption/ionization (MALDI) mass spectrometry.

## Supplementary Information

Below is the link to the electronic supplementary material.

Supplementary file1 (DOCX 4798 KB)
