# Supplementary material for: Introduction of Octadecyl-Bonded Porous Particles in 3D-Printed Transparent Housings with Multiple Outlets
Source: Chromatographia. 2022 Jun 22;85(8):783–93. doi: 10.1007/s10337-022-04156-w (PMC9363280; doi:10.1007/s10337-022-04156-w)
Supplement: Supplementary file 1 — Supplementary file1 (DOCX 4798 KB) [file 10337_2022_4156_MOESM1_ESM.docx]

Introduction of octacedyl-bonded porous particles in 3D-printed transparent housings with multiple outlets

Liana S. Roca^1^, Theodora Adamopoulou^1^, Suhas Nawada^1^, Peter J. Schoenmakers^1^

1. Van ’t Hoff Institute for Molecular Sciences, Science Park 904, 1098 XH Amsterdam, Netherlands

Supplementary Information

1. Device design and printing


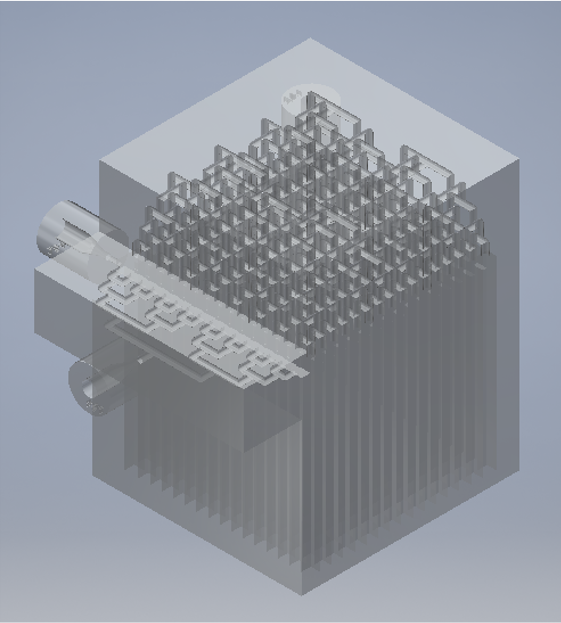


Figure S1 CAD design of a 3D separation device with flow distributers in the second and third dimension


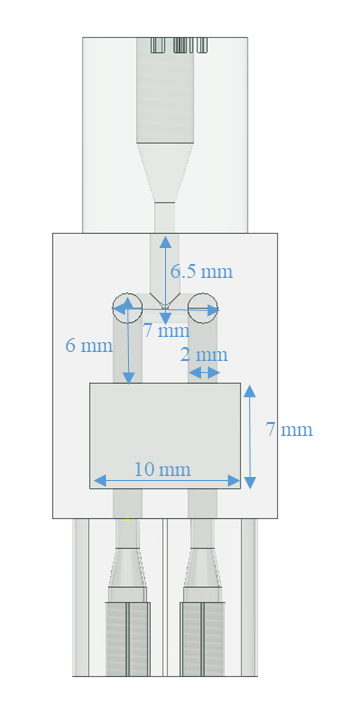


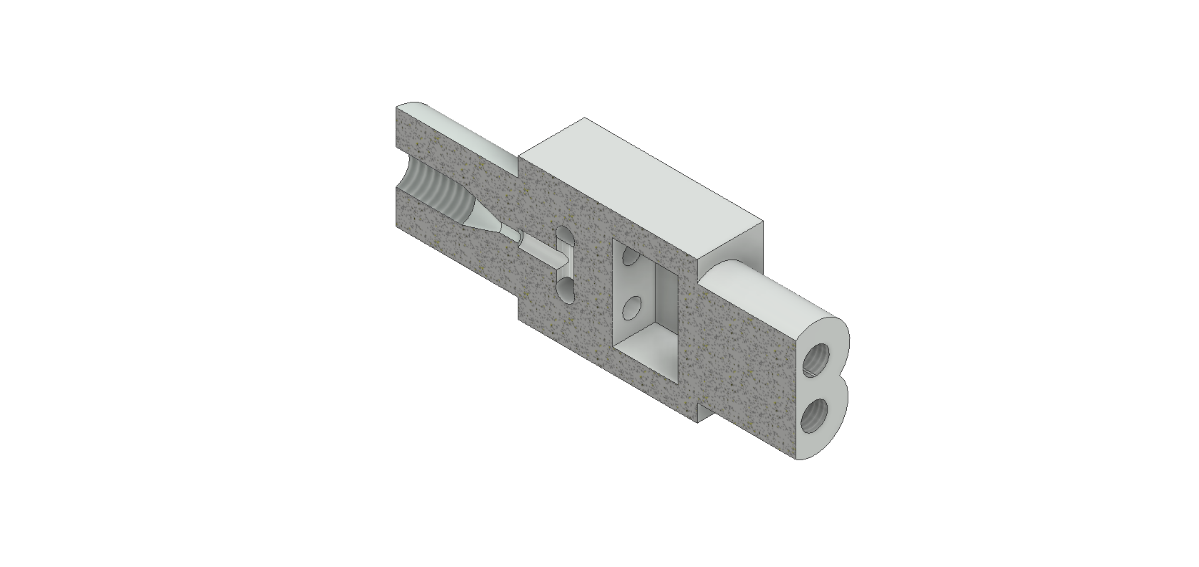

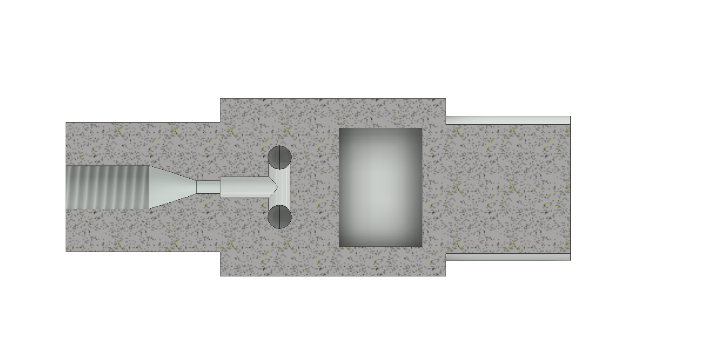


Figure S2 Drawing of the 3D-printed device with dimensions (left) and a cutaway through the middle of the device (right) showing the design of the flow distributor (top), the separation space (middle) and the outlets (bottom).


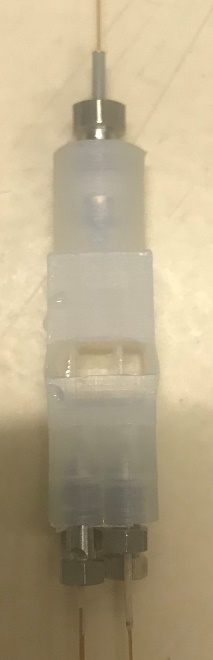


Figure S3 1 mm wall thickness, device broke at 35 bar around the separation space


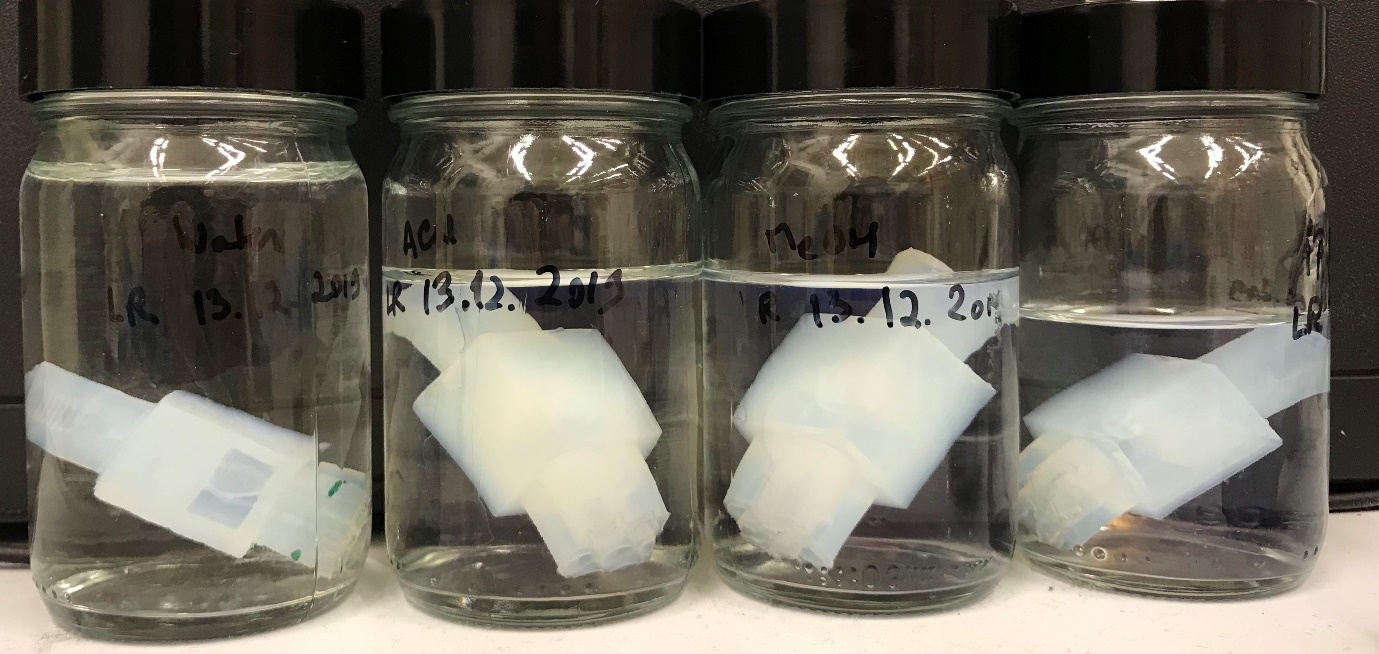


Figure S4 Devices after 24 h in different solvents. From left to right; water, acetonitrile, methanol, iso-propanol. The most affected device was in methanol followed by acetonitrile and iso-propanol. The device in water was unchanged.

1. Packing of devices


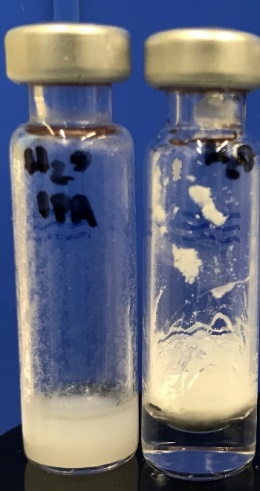


Figure S5 C18 particles suspended in 50% IPA (left) and in water (right). The C18 particles are hydrophobic, hence no solvation is observed. By the addition of 50% IPA solvation was possible.


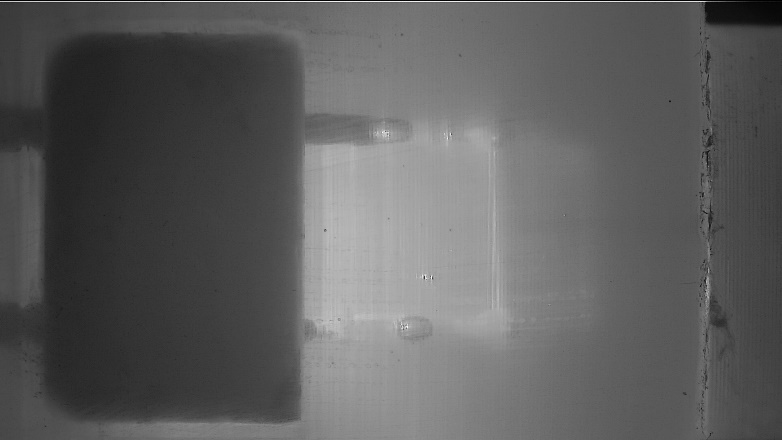

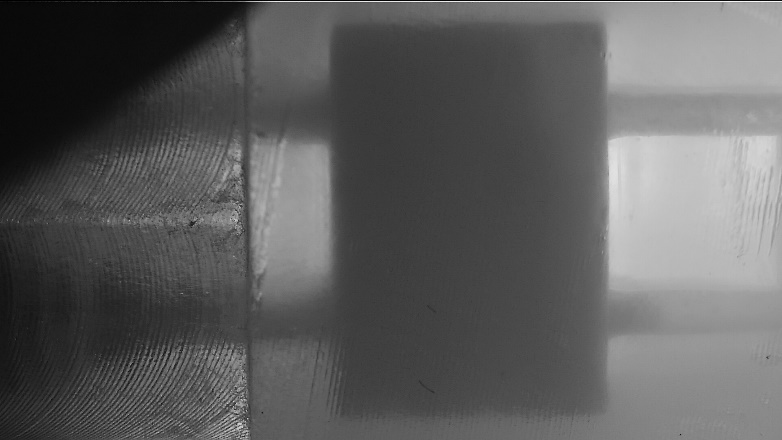


Figure S6 Importance of flushing and sonication in IPA before curing. Left image shows rounded corners of the cube while the picture on the right has sharp edges. Residual resin can solidify in the UV curing oven if the device is not cleaned before.


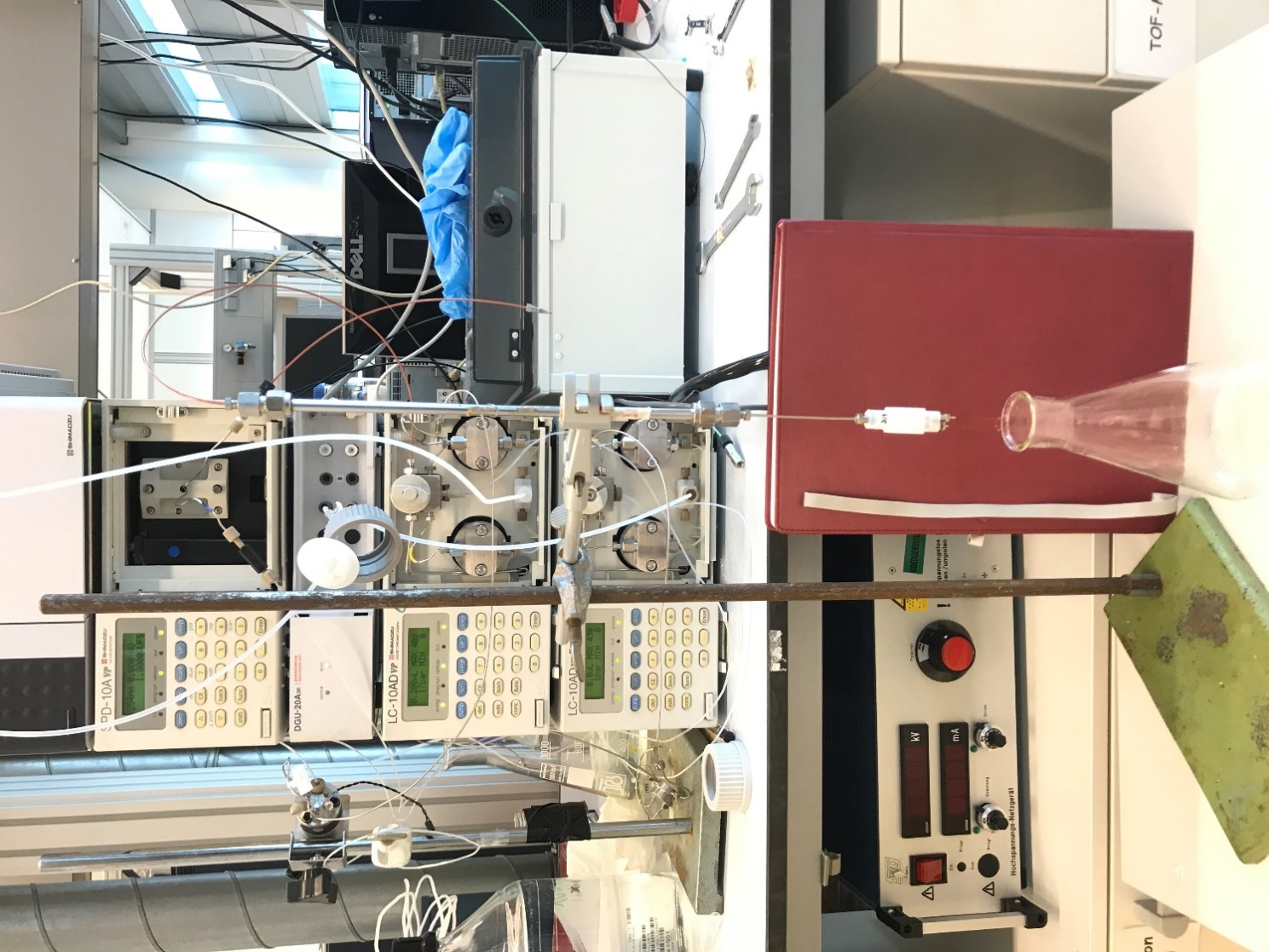

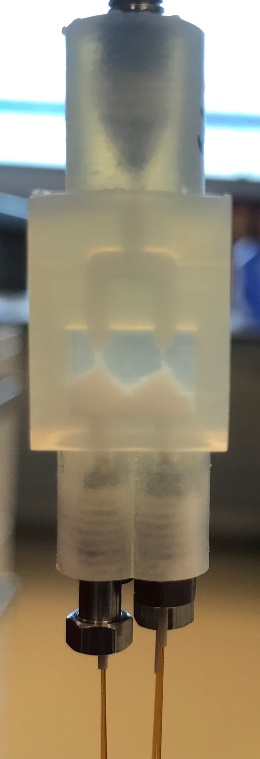


*Figure S7 Vertical alignment of the packing setup during the introduction of the C18 particles.*


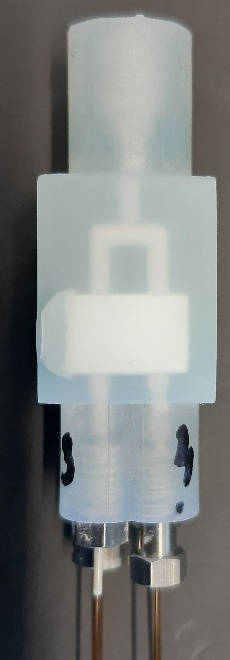


Figure S8 Device that broke during packing. The break can be observed above outlet 3 on the edge of the separation space. A possible explanation is clogging of the outlets during packing that caused the pressure to increase to the point of breaking.

1. Characterization of packed devices

Table S1 Influence of the packed flow distributor on the device backpressure, where section 1 Is the FD inlet channel, Section 2 is the first bifurcation, Section 3 second bifurcation and section 4 are the 4 channels connected to the separation space.

|  | Separation Channel | Flow distributor | | | | Exits |
| --- | --- | --- | --- | --- | --- | --- |
|  |  | Section 1 | Section 2 | Section 3 | Section 4 |  |
| Number of parallel channels |  | 1 | 2 | 4 | 4 | 4 |
|  |  |  |  |  |  |  |
| Diameter (mm) | 10 | 2 | 2 | 2 | 2 | 2 |
| Area | 100 | 3.14 | 6.28 | 12.57 | 12.57 | 12.57 |
| Relative linear velocity (total flow 100) | 1 | 31.83 | 15.92 | 7.96 | 7.96 | 7.96 |
| Length | 7 | 6.5 | 3.5 | 3.5 | 6 | 2 |
|  |  |  |  |  |  |  |
| Relative pressure | 7 | 206.90 | 55.70 | 27.85 | 47.75 | 15.92 |
|  | 2% | 57% | 15% | 8% | 13% | 4% |
|  | 2% | 94% | | | | 4% |

- 1. Packing with sonication

Figure S9 Flow output was collected from all outlets and weighed. Measurements were performed in triplicate and the average value was plotted with the standard deviation. 25% in each outlet would be an ideal situation

Figure S10 The average permeability was calculated using three flow-rates (0.1, 0.2 and 0.4 mL/min)

3.2 MS detection


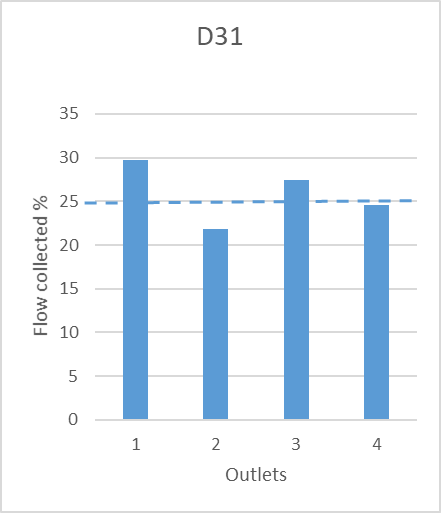

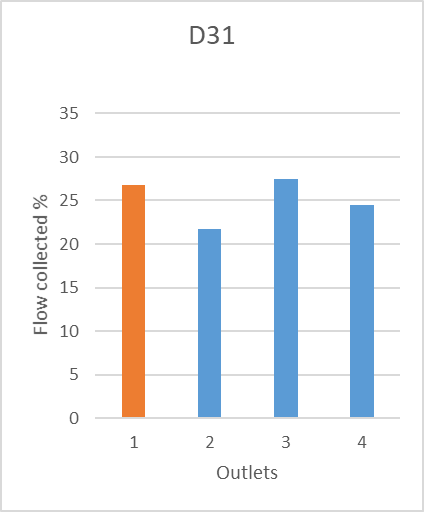


Figure S11 The flow output measurement of the 4 outlets can be seen in the left graph and the flow output with outlet 1 connected to the MS can be seen in the graph on the right. The connection to the MS source had no influence on the backpressure of the device or the flow output.

Table S2 Flow collected before and after connection of outlet 1 to the MS. The measurements were repeated 3 times and the average was reported. Theoretical weight of the combined effluent should be 1.89 g.

| D31 | Free outlets | | Outlet 1 connected to MS | |
| --- | --- | --- | --- | --- |
| Outlet | % collected | Effluent collected (g) | % collected | Effluent collected (g) |
| 1 | 30 | 0.5602 | 26 (expected) | - |
| 2 | 22 | 0.4095 | 22 | 0.4122 |
| 3 | 27 | 0.5162 | 27 | 0.5213 |
| 4 | 25 | 0.4603 | 25 | 0.4650 |
| Sum | 104 | 1.9462 | 100 | 1.3985 |


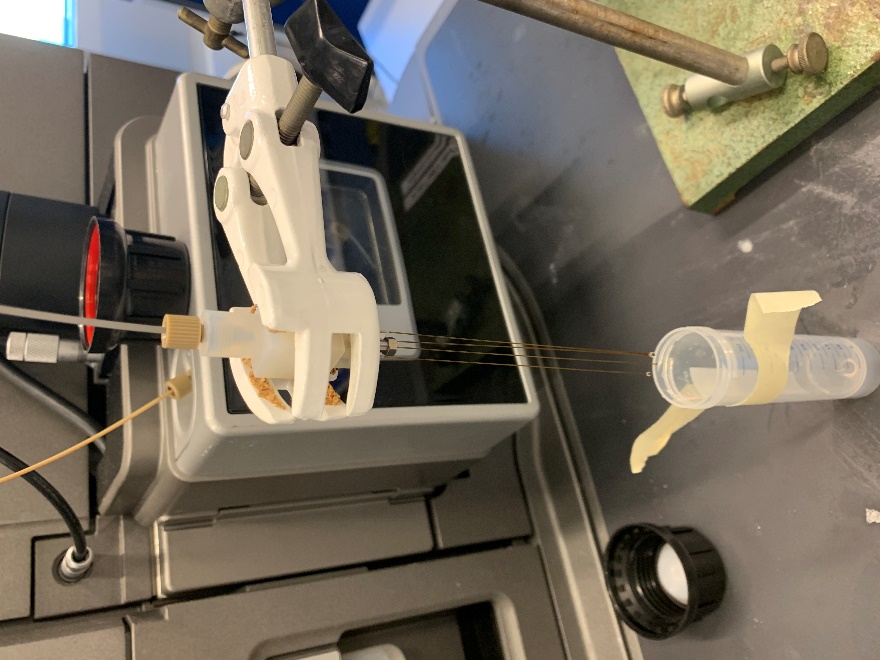

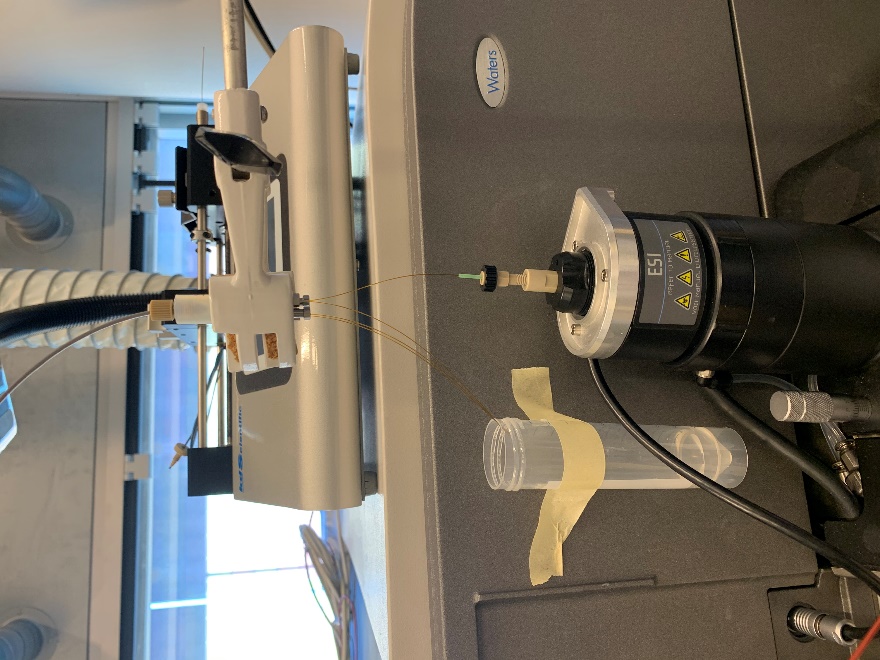


Figure S12 The flow from each (free) outlet was measured before (left picture) and after (right picture) connection of outlet 1 to the MS. The connection was by adding a sleeve and a PEEK nut to the outlet capillary, and further connected to the MS ESI source.

*Figure S13 Structure of the peptides used to perform RPLC separation with the packed device.*





Figure S14 Triplicate measurement of gradient separation of peptides in outlet 4. The Extracted ion currents were overlaid. Order of elution was Gly-Tyr, Val-Tyr-Val, Met-Enkephalin and Leu- Enkephalin.
